# An Advanced Algorithm for Higher Network Navigation in Social Internet of Things Using Small-World Networks

**DOI:** 10.3390/s19092007

**Published:** 2019-04-29

**Authors:** Farhan Amin, Rashid Abbasi, Abdul Rehman, Gyu Sang Choi

**Affiliations:** 1 Department of Information and Communication Engineering, Yeungnam University, Gyeongsan 38541, Korea; farhan@ynu.ac.kr or farhanamin10@hotmail.com; 2 School of Computer and Technology, Anhui University, Hefei 230039, China; rashidd.abbasi@gmail.com; 3 Department of Computer Science and Engineering, Kyungpook National University, Daegu 41566, Korea; a.rehman.iiui@gmail.com

**Keywords:** link selection, Internet of Things, Social Internet of Things, network navigability, small world

## Abstract

The Internet of Things (IoT) is a recent evolutionary technology that has been the primary focus of researchers for the last two decades. In the IoT, an enormous number of objects are connected together using diverse communications protocols. As a result of this massive object connectivity, a search for the exact service from an object is difficult, and hence the issue of scalability arises. In order to resolve this issue, the idea of integrating the social networking concept into the IoT, generally referred as the Social Internet of Things (SIoT) was introduced. The SIoT is gaining popularity and attracting the attention of the research community due to its flexible and spacious nature. In the SIoT, objects have the ability to find a desired service in a distributed manner by using their neighbors. Although the SIoT technique has been proven to be efficient, heterogeneous devices are growing so exponentially that problems can exist in the search for the right object or service from a huge number of devices. In order to better analyze the performance of services in an SIoT domain, there is a need to impose a certain set of rules on these objects. Our novel contribution in this study is to address the link selection problem in the SIoT by proposing an algorithm that follows the key properties of navigability in small-world networks, such as clustering coefficients, path lengths, and giant components. Our algorithm empowers object navigability in the SIoT by restricting the number of connections for objects, eliminating old links or having fewer connections. We performed an extensive series of experiments by using real network data sets from social networking sites like Brightkite and Facebook. The expected results demonstrate that our algorithm is efficient, especially in terms of reducing path length and increasing the average clustering coefficient. Finally, it reflects overall results in terms of achieving easier network navigation. Our algorithm can easily be applied to a single node or even an entire network.

## 1. Introduction

The Internet of Things (IoT) is a technology that connects a huge number of homogeneous and heterogeneous objects. These objects have the ability to communicate and to provide a number of services. These objects are also known as smart objects [1,2], as they continuously generate information about the physical world. Additionally, they could be anything: a small sensor or a general multipurpose computer. The information received from smart objects is accessed through several platforms and standard web servers. These web servers offer an application programming interface (API) for the data retrieved from actuators and sensors [1]. 

Consequently, by using IoT-based technologies it is possible to provide various services to the connected end-users in many fields, such as city management, home management, environmental monitoring, or industrial monitoring [3]. Due to technology improvements, attached devices are getting smarter. According to a recent survey, the number of devices connected to the internet will exceed 30 billion by the year 2020 [4,5]. Therefore, searching for a service in IoT devices appears to be a challenge [1]. Since a device can have numerous connections, it therefore requires a large search space in order to find something in the entire network [5]. According to a study by Nitti et al. [1], after 2015 the number of radio frequency ID (RFID) devices will reach hundreds of billions, and hence search results will increase network traffic. As search engines receive a number of queries simultaneously, it is very difficult to manage and handle them efficiently by using currently available platforms. From this perspective, there are several algorithms proposed for real-time systems [6,7]. One of the common features of these approaches is to focus on centralized systems, and hence they cannot properly scale up to the huge number of devices or receive several queries. In order to address and handle the scalability issue of centralized systems, a new paradigm was introduced, called the Social Internet of Things (SIoT) [8]. The SIoT is the addition of social networking to the IoT. Moreover, social networking is limited not only to online social networking websites like Facebook and Twitter, but it can also be applied to the natural sciences. The communications among these objects is improved by using smart objects, especially when they are connected to others. These smart objects have the ability to communicate directly or indirectly by utilizing their neighbors. One of the key features of the SIoT is that every object can look for a desired service by using neighborhood relations, such as querying neighbors or neighbors of neighbors, in a distributed manner. These objects directly communicate with other objects, as demonstrated in the above scenario. These friendship links are established by using a distributed approach. A friend request is sent to all nodes in a network without connecting to a centralized search engine. Usually, in SIoT-based networks, every node is an object and is capable of establishing social relations with others in an autonomous way according to the rules set by the owner [1]. In the SIoT, objects become socially connected and smart [9]. Accordingly, they can provide several benefits: (i) they interact with other objects independently with respect to their creator or owner [9]; (ii) they have the ability to advertise their presence in the network, and this later results in providing a number of services; and (iii) these objects are mobile, and hence can move easily anywhere in the network [10]. Thus, we conclude that billions of objects in the IoT have the ability to discover and disseminate information and services in a trustworthy manner [9]. Additionally, these objects have the ability to collaborate with other objects in order to achieve a common objective. The assumption is that the network will be navigable and based on the principle stated by the famous sociologist Milligram about his study of small-world problems [11]. The term ‘small world’ includes a short chain in the existence of small chains among individuals in societies [12]. Starting from the Milligram experiment, Kleinberg concluded that there are a few structural clues that can help people to find a short path efficiently, even without global knowledge of the network [13]. Each object can store and manage the information related to friends, implement search functions, and eventually employ some additional features, such as trustworthiness, that are useful in evaluating the reliability of a friend [14]. In a network, various connections affects the consumption of memory and computational power. In addition, a service search depends upon a number of links between objects.

The motivation of our study is to find the desired service for objects in the IoT. In IoT, objects usually reach out to neighbor objects in a decentralized manner to look up a service. The search procedure goes through friends and friends of friends in a distributed manner. It is almost impossible to make all objects into friends, because an object needs to store and manage information about its neighborhood. We already know that IoT devices have limited memory and computational capabilities, so it is difficult to store information about a number of attached devices. In order to provide a service to these devices, we need to carefully think about the number of devices attached to a device, which should be chosen carefully and should be minimal. It has already been discussed in the literature that the selection of neighbors for any device should bring reachability to other devices. The selection of friends in the IoT was discussed [1,15]. By taking care of network navigability, this study analyzes a few strategies to be implemented by each node when the node wishes to add new nodes. Our proposed algorithm addresses some limitations in existing strategies. The major contributions of our study are as follows. 

(1) We address the problem of link selection by restricting the number of connections in a single node by using a threshold, ‘r’, in order to increase the network navigability. 

(2) We analyze the behavior of existing strategies and the routing performance of objects by exploiting local information about links, referred to as degrees. In this way, each node is not obliged to have the local network topology, i.e., reducing routing complexity. Then, we discovered that if we try to minimize local clustering in the network, it will be beneficial in the achievement of best results in terms of getting a small path length. The identified number of connections (hubs) is the motivation for this discovery. Accordingly, we propose an advanced algorithm having a threshold value, and can be adjusted dynamically on the basis of the number of hubs in a network. In this way, the degree distribution is closer to the power law, and we are able to guarantee local network navigability.

(3) In existing studies, the authors focused on preferential attachment described by Barabasi–Albert (BA) model [16]. However, this model generates smaller clustering coefficient. Hence, in our study, we have used a modified version of the HK model known as power law [17] The power law supports an extra step called triadic formation, which results in the achievement of larger clustering coefficient, and is later helpful in getting shorten paths between nodes in a network. Finally, it reflects the overall network, resulting in attaining of higher network navigability.

(4) We propose a link selection algorithm for the selection of appropriate links in a network. Our algorithm is completely based on local network properties of small-world networks, such as local clustering and neighborhood degrees. Our algorithm is used to rank the nodes in decreasing order and to choose the ones that maximize the chosen algorithm. Its performance was analyzed in terms of the giant component, the average path length, and the average clustering coefficient. 

This paper is organized as follows. In Section 2, we present a review of service searches in the IoT and the SIoT, we introduce a reference scenario for distributed search, and discuss current problems. The current state of the art in network navigability and small-world networks is discussed in Section 3. Section 4 describes the selection of network links from our proposed work. Section 5 describes an experimental evaluation of our study, and Section 6 concludes the study with final remarks and future work.

## 2. Background and Related Work

### 2.1. The SIoT and Service Search in the IoT

The SIoT can be described in terms of the IoT [8]. In the IoT, independent objects connect to each other by using certain links. The connected objects may be anything, such as a computer or a user. In the past, cloud computing was used to provide services to a number of users. The term ‘cloud’ computing refers to access to the computing resources across a network [18]. A social cloud is defined as a resource- and service-sharing framework utilizing relationships established between the members of a social network. These resources are not limited to networks, and include storage, servers, and services. Cloud computing and social networking have intermingled in a variety of ways. One of the advantages of the social cloud is scalability; typically, social clouds provide low-level abstraction of computation or storage [18]. The benefit from its use is optional, as users may or may not wish to share resources. Chard et al. designed a prototype of the social cloud [18], and their prototype is based on a credit model. The credit model is used to regulate and exchange by preventing freeloading. Their model is purely based on economic exchange and emphasizes user choice. Hence, their model only fits from the perspective of selling resources using a social cloud, which is not a key aspect of the social cloud [19]. So, Chard [20] presented and deployed a method for Facebook, using a social storage cloud. The storage cloud supports storage trading by using two protocols. The integrated storage of the Facebook application allows users to discover and trade the storage contributed by their friends, and takes advantage of pre-existing trust-based relationships. In order to handle trust relations, they developed a credit-based trading approach to discourage freeloading. Their proposed model is similar to the self-organization cloud. However, if the users in that organization are anonymous, and hence cannot account for their actions, then accountability can be established by using existing friend relations [21]. Hence, the social cloud is sometimes not an effective solution for service discovery, due to connection dependency, loss of control, slow connections, etc. Thus, finding a service for objects and making relations turns to other solutions, such as machine-to-machine (M2M) communications.

M2M refers to direct communications between devices using any communications channel, either wireless or wired. The Example applications are smart grids or smart meters presented by Tsiropoulou [22]. In these applications, objects usually make a point-to-point (P2P) connection for communication. One of the benefits of M2M communication is to provide good connectivity between large number of low-cost devices with the least amount of human intervention [22]. According to a recent survey, more than 100 billion IoT devices are connected with each other worldwide. By 2025, this number will increase (i.e., an addition of more than US$11 trillion [23]). Therefore, owing to this huge number of connected devices, several issues arise, such as energy efficiency and wireless access congestion [22]. In order to solve these problems in parallel, and improve energy efficiency, numerous joint clustering schemes have been proposed. These methods are mainly based on resource management and the interconnection of devices [23], or simply known as communication of interest (CoI). According to a recent study [22], numerous M2M methods have been proposed in the past and are based on different criteria, such as M2M achievable signal-to-interference-plus-noise ratio (SINR). There are a few studies in which data priority was proposed [22]. Data priority is the flow of data from a specific M2M device. The priority is set based on the transmitted and received data. The collected data have a higher priority than received data. It has been observed in many cases that joint consideration of cluster formation and power management demonstrates better results [14]. The notion of communication interest among users is also helpful in creating homogeneous coalitions and was discussed by Tsiropoulou et al. [22]. 

In that study, they discussed a coalition formation framework that is based on the problem of energy, on interests, and that can be used for resource distribution among IoT applications. They assigned a holistic, utility-based function to a number of attached IoT devices. This function appropriately represents the degree of satisfaction with respect to QoS. Their framework is divided into two stages. In the first stage, a time-based slot is assigned and a coalition head is determined [14]. In the second stage, determining QoS prerequisites of M2M devices is performed with a prior holistic utility function. The holistic function enables this framework to be used within different IoT applications due to its generic form. The framework is energy-efficient and can be used for the coordination of many devices. However, there is no security nor privacy-based criteria discussed by Tsiropoulou [22]. In [23], Tsiropoulou and colleagues suggested a low complexity coalition formation mechanism among IoT devices based on the Chinese Restaurant Process (CRP). The devices in the CRP can harvest energy from RF signals and based on wireless powered communication (WPC) [23]. This approach enables the use of clustering with the help of cluster heads and members. Their approach can harvest and stably store energy via RF signals, by adopting the wireless rechargeable sensor network paradigm. There is no real testbed used in this framework. Additionally, there is no discussion related to a security paradigm [23]. The issues of a single point of failure in cluster heads and energy harvesting still persist in cluster-based schemes. So, in order to handle these issues, considering the link selection algorithms as a remedy in the social IoT is a good idea. 

**Figure 1:**
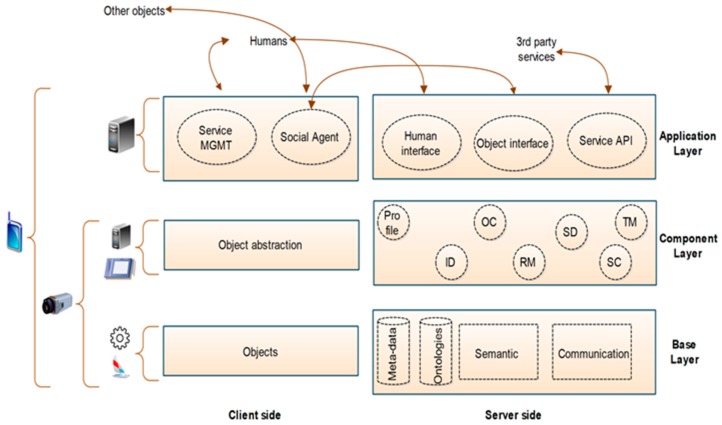
The basic architecture for the SIoT.

We refer readers to a recent study based on the social IoT [1]. In this model, the authors have explained the basic architecture for the SIoT, as shown in Figure 1. The SIoT model is divided into three major layers: base, component, and application [24]. The first layer is used to provide various services, such as databases, communications services, semantic engine services, etc. The middle layer is the component layer and is used for satellite component implementation. The application layer acts as an interface between humans and objects. Furthermore, it provides connection to the services. On the left-hand side, the client-side module is divided into three layers: objects, object abstractions, and a social agent. The objects layer comprises a collection of physical objects, and acts as an interface between attached devices. The interface is controlled by using some common programming languages. The upper layer is comprised of a number of agents, and the main function is to make new connections between the attached objects and the social IoT. The service management layer provides two types of service: the first one is an interface, and second monitors and controls the behavior of objects. The benefit of this proposed model is to overcome various issues such as service composition and service discovery. 

One of the benefits of the SIoT to allow objects to make new links in a network. It is mainly based on the social relationships among the objects and has become popular in the last few years [1]. Our aim is to explore social networking and the discovery of numerous features, such as selection, discovery, and composition. We already know that information is mostly provided to distributed objects and networks are accessed from the physical world [25,26]. There are some real-world examples in which various authors have explained the role of the service search in a network. For example, studies deal with the representation of many objects by using a hierarchy of intermediator nodes [7,27]. These nodes are usually smaller and are responsible for the grouping of data in certain terrestrial areas. A single mediator node is put at the top level, and will keep an aggregated view of the whole network. These solutions are not good regarding scalability or in the case of a frequent change in data rate [28]. They work well, especially with static metadata. an centralized prediction-based model was proposed by Tie-li in [6], and calculations were performed based on the probability of a matching query. The advantage of this method is that the search engine does not need to contact all of the sensors available in a network. Hence, it results in the achievement of good scalability [29]. Due to the increases in sizes of networks and in the number of objects in a social network, service selection is going to be a difficult task, since an object is connected to millions of other objects in a network in a heterogeneous manner [29]. Hence, the service search becomes difficult. The search service in the SIoT is carried out using a two-step procedure: first, by performing some sensing procedure, and second, to retrieve the data, which may reside on other devices. The requirements to search for an object is divided into two main types: proximity and point [30]. In the first type, the search intention of a particular device is performed (for example, monitoring the temperature at a fixed location). In proximity-based requirements, the search procedure is quite flexible, and can be carried out by using certain variations. The advantage of this method is that a user may tolerate a small variation while looking for a specific service. 

**Figure 2:**
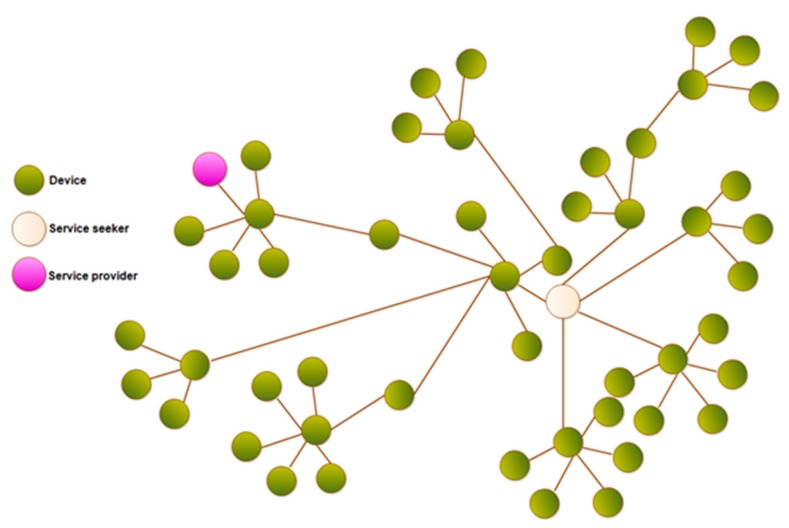
Searching for services in the IoT.

In the IoT, usually objects look for services provided by other objects in a decentralized manner, as shown in Figure 2. The search procedure is initiated by looking into the neighbor devices in an intelligent manner. In this figure, the attached devices are represented in green; orange indicates a service seeker and pink indicates a service provider. In this picture, we clearly observe a number of devices attached between information seeker and service provider device. Additionally, we do not know about the number of hops between the information seeker and information provider. Hence, it is difficult to connect all devices between information seeker and information provider, and to make several links between them. The question is why not all devices? The answer was addressed in an earlier study [30], since a service seeker has the ability to save necessary information about its neighbors. However, we already know that IoT devices have limited memory and computational power; hence, it is technically impossible to save all information related to neighbors. Additionally, achieving feature-based variations among neighbors is still difficult, because one device in an IoT network holds data and information for several neighbors. Hence, the criteria should be based on the selection of a minimal number of neighbors per device. If we choose the least number of devices for one device, then it will definitely increase the reachability of a device to its nearest neighbors. So, in this study, we explained how to achieve higher network navigability by restricting a node to the least number of connections per device in a network.

### 2.2. Distributed Search Referenced Scenario

There are different ways to search the contents of the internet, such as searching for a web page or video. This procedure is very similar to the search for data from objects and sensors in the IoT. In the near future, it is expected to be a major service area in the IoT. In the social IoT, objects inherit some experiences of human behavior when they look for new services or friends [1]. In this network, an owner usually sets certain rules and each object can create and manage several types of relationships with itself and its neighbors [31]. Additionally, an object has the ability to ask friends about providing services.

**Figure 3:**
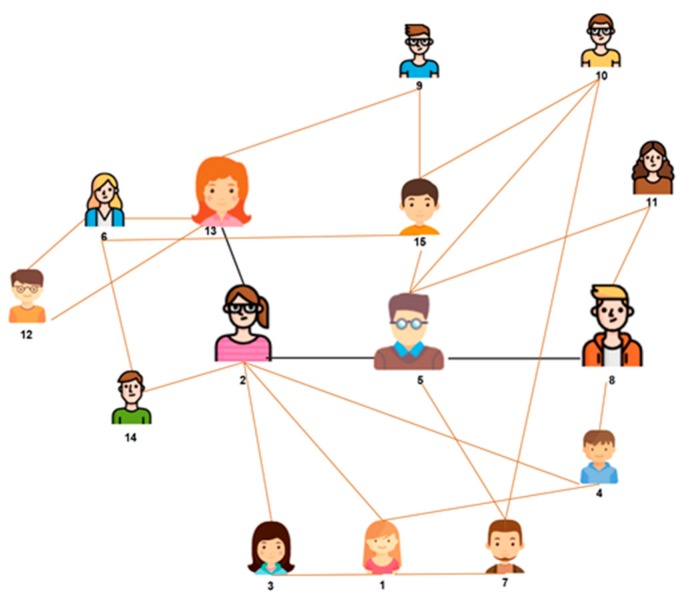
Search for services: a reference scenario.

Figure 3 provides an exemplary scenario of a decentralized search and illustrates a search mechanism. In this figure, links represent friendship ties and bold lines describe the best route. We considered an IoT network as a visualized graph, and individual nodes are represented as friends. The edges between friends are referred to as links. Some friends available in this network are higher in degree, and are represented at a bigger size (e.g., 8, 5, 2, and 13), while the rest of the friends are smaller because they have a lower degree.

In this figure, friends are numbered from 1 to 15. Suppose, a service seeker named friend 8 is looking for a service, and it can be assumed that the required service resides on service provider 13 (the service seeker, i.e., friend 8, does not know about the service provider), when an information seeker requests a particular service, it does not need to send a request to a centralized search engine. Usually, it uses its own first-hop friends and then looks for that service in a decentralized manner. 

The search procedure for the information seeker is described as follows. In the first step, the information seeker will search for a service among its neighborhood circle, such as friends 4, 5, and 11. The property will guide the information seeker to select friend 5 as the next hop; it has a high degree of centrality. So, friend 5 is selected because it represents a network hub (this friend is connected to many friends). The ability for a friend to reach a network hub is assured by the existence of network clusters where friends are highly interlinked. This feature is assured by using a high value for clustering. The same procedure is repeated for the selection of the second hop, i.e., friends 2, 7, etc. Thus, friend 2 is selected. Finally, friend 2 checks its next-hop neighbors, i.e., friends 14 and 13. By repeating the same procedure, finally, the information provider is selected. Hence, the search procedure is over and a permanent link is created between information seeker and information provider, via the friendship circle, i.e., 8, 5, 2, and 13. 

In this section, our aim is to evaluate the effect of the current distributed search procedure in the SIoT. In the next few paragraphs, we highlight and explain the importance of network navigability with the help of real examples and a few studies in detail. 

## 3. Network Navigability in Small-World Networks

Network navigability is not new; it has been widely studied in the past, especially in small-world networks [32] proposed by Kleinberg et al. [13]. A small-world network is navigable if there exists some short route to connect all pairs of nodes in the network. Kleinberg et al. presented the concept of an infinite family of small-world networks that generalizes the Watts and Strogatz model [32]. The former demonstrated that decentralized search algorithms are helpful in finding short paths with a high probability. Usually, nodes in a network have the ability to make new connections by using their neighbors. One of the advantages of network navigability is global knowledge of the network, in which each node has full information about network connections [33]. Another condition for network navigability is the existence of a giant component in which a lot of nodes are connected. 

Consider a network where each node has complete information about global network connectivity. The distance between any pair of nodes should not exceed log_2_ (N), where N is the number of nodes. In this network, a search of short communication paths is only a matter of distributed computation. Hence, this solution is not practised as these should be applicable to a centralized engine. It might be possible for a friend node to handle a request from neighbors by using local network navigability, in which nodes need to communicate with each other by an exchange of information. Hence, the problem of global network navigability turns into local network navigability. Once, studying Travers and Milgram’s [34] and Kleinberg’s [13] experiments, we observed that both of them have the same structure. These experiments are helpful for many people who intend to find the shortest paths in a network, even without the global knowledge of the entire network. We then concluded from the above studies that properties of social networks make the distributed search better. In the next few paragraphs, we will discuss some new heuristics for attaining high network navigability.

### 3.1. Discussion of the Current State of the Art in Network Navigability

The objective of this study is to restrict the number of friends currently accessed by a network, and to try to increase network navigability. Our intent is to increase reachability and to make shorter links. Instead of using and traversing longer paths, shorter paths will reduce computer processing, and make a search process faster. Additionally, the available friendship circle enhances the rise of network navigability, and this will provide global information about the entire network, in which each node uses only local information for access to services. The key properties of an object’s navigation in a small-world network are as follows. 

#### 3.1.1. Average Degree 

The degree of a network is represented by measuring the number of friends for a node. The average degree is measured by taking the ratio for the number of degrees of each node to the total number of nodes in network. It is observed that average degree is increased by growing the number of relations inside of the nodes.

#### 3.1.2. Average Clustering Coefficient

This was introduced by Watts and Strogatz [32], and is a measure of the closeness of nodes in forming a clique. It is calculated by each node in a network, and can range from 0 to 1. The clustering coefficient for an undirected and a directed network differs, and is written separately, as shown below. 

For an undirected network:

(1) $$C_{n}=\frac{2e_{n}}{\left(k_{n}\left(k_{n}-1\right)\right)}$$

For a directed network:

(2) $$C_{n}=\frac{e_{n}}{\left(k_{n}\left(k_{n}-1\right)\right)}$$

where *k_n_* represents the number of connections and *e_n_* represents connected pairs between neighbors [35].

The average clustering coefficient for all nodes in a network is

(3) $$\bar{C}=\frac{1}{n}\overset{n}{\underset{i=0}{\sum }}C_{i}$$

In Equation (3), *n* represents the number of nodes and $C_{i}$ represents local clustering. Additionally, local clustering for each node is computed separately. The algorithm is considered efficient, and tries to achieve higher values for clustering. 

#### 3.1.3. Average Path Length 

The path length is represented by calculating the shortest distance between any two nodes in a network. An efficient algorithm always tries to find a low average path length.

#### 3.1.4. Giant Component 

Usually, a group of nodes or the percentage of numerous nodes connected directly or indirectly in a graph, is called a giant component. The giant component will be increased by increasing the number of links in a network. It results in high network navigability. It is observed in many cases that if a network has a higher giant component, it definitely has higher network navigability. If the giant component is 100%, this means that all nodes are reachable by all other nodes in the network.

The heuristics proposed in earlier research [5,15] are given below. These heuristics try to achieve some sort of tradeoff between several metrics discussed in the above section.

(a) Reject after N_max_: The objective of this strategy is to make static friends. A unique threshold, N_max_, is assigned and the decision to accept or reject is purely based on N_max_.

(b) Maximize Neighborhood: If a new incoming request is made, and the node has already reached N_max_, the node will immediately delete a friend with a minimum degree to make room for the incoming request. 

(c) Minimize Neighborhood: Once a threshold value is reached and an incoming request is made, this strategy will find a node that has the maximum degree and will delete it immediately. 

(d) Maximize clustering: Once N_max_ is reached and a new incoming request is made, this strategy will find a node with a minimum number of mutual friends and eliminates it. 

(e) Minimize clustering: If a new request is made that reaches N_max_, this strategy selects a friend that has the maximum number of mutual friends and later eliminates that friend. 

We carefully performed an analysis by using five rules described in Arjunasamy et al. [5] and Nitti et. al. in [15]. Maximum local clustering is achieved when strategy (d) is applied. However, it suffers from a low percentage in the giant component. We already know that the presence of a low giant component affects a service search such that search efficiency is reduced. Furthermore, it was observed in many cases that it results in attaining a high clustering level. 

These strategies still lack a few aspects, such as how to provide information to objects, and how to build a reliable system. Hence, our proposed algorithm is helpful in reducing the number of connections per node, in eliminating old mutual friends, and in making a new connection to a node that has a high number of connections.

The proposed algorithm is discussed in the next section. 

## 4. Selection of Network links

Our proposed algorithm addressed some limitations in current heuristics, and therefore we suggest a new solution that works as a remedy. The explanation of our algorithm is given below.

### Explanation

At the beginning of the Algorithm 1, the search for a friend is performed by looking into the owner’s list, i.e., Node N_b_ is looking for service amongst its neighbors. During this lookup procedure, the degree of each node is computed separately. We restricted r to a maximum number of neighbors for each node, such as r = 5 in our list. 

**Algorithm 1. Link Selection.****Input:**Send a friend request**Output:**Obtain Friendship circle “F”Start ()
 **Step 1:****Find a friend**  If {P(k) of node N_b_ <= r}   {Decide mutual friends; M_(x,y)_ of node N_b_ with minimum mutual friends}   {Remove node Nc from the list}   {Add node Nr to the list for hop12}  Else If {P(k) of node Nc = =1}   {Decide neighbor friends of Nb with minimum P(k)}   {Add node Nc to the list of Nb for hop12} **Step 2:**
   {Decide eldest friend from neighbors of node Nb with minimum P(k)}   {Remove node Np from the list} **Step 3:**
   {Decide eldest friend from neighbors of node; i.e. Nb as Np}   If {P(k) of node Np = =1}   {Search in first two-hop friends (N_hop12_) of node Nb and P(k)< r;}   {Add node Np to the friend list of Nr_hop12_} **Step 4:**
   {Decide friend with least priority among all friends of node Nb}   {Eliminate node Nq from list}**End ( )**


In Step 1, the search procedure for a new link is initiated, and the degree of each node is computed until the friend count reaching r is finished. This is used to achieve the triadic closure. After that, if the number of edges exceeds r, it simply refuses the incoming request; otherwise, the search in the neighborhood for mutual friends continues. A minimum mutual friend node is selected by using Equation (4):

(4) $$M_{\left(x,y\right)}=\left|N\left(X\right)\right|\left|N\left(Y\right)\right|$$

After that, it simply removes N_r_ from the list. If the selected mutual friend N_c_ has a degree of zero, it simply elimnates the old link and establishes a new link by using second-hop friends. In Step 2, the search for the eldest friend is performed by looking into the first-hop neighbors; if successful, the eldest friend will be eliminated. Our aim is to handle unexpected situations that arise, especially in the case of a mobile device. Usually, mobile devices have a common mobility issue, since they are not fixed in one place. So, our algorithm eliminates mobile mutual friends from the list and makes new connections. 

This step is suitable for scale-free networks since we try to improve the object navigability in the social network.

We will explain in the next few paragraphs how our proposed algorithm works with the help of an example scenario.

#### Example Scenario 

**Figure 4:**
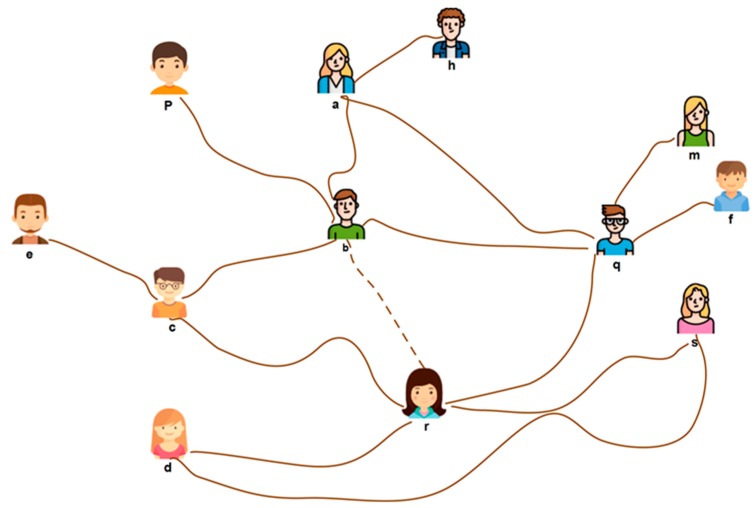
Selection of Network links.

Let us consider a network of friends, N = {N_a_-N_q_}, is connected to each other as described in Figure 4. Suppose a service seeker, node N_r_, is looking for a service and sends a friend request to node N_b_. The dotted line represents a friend request between node N_r_ and N_b_, as shown in Figure 4. 

In order to accept the request, node N_b_ implements our algorithm. If the incoming request node, N_r_, is higher in degree, and node N_b_ has not reached r, then N_b_ finds a friend that has a minimum number of mutual friends. This results in the selection of two nodes, such as N_c_ and N_q_ in the owner’s list. Node N_r_ compares its own degree with nodes N_c_ and N_q_. That results in the elimination of N_c_ from the list, and in addition, creates a new node, N_r_, on the owner’s list. If node N_c_ has a degree of zero, node N_c_ will be suggested to one of the friends or the friend of a friend of node N_b_, as a new link. In this example, it has no effect, and hence, a friendship circle is established between nodes N_r_ and N_b._

In the second step; node N_b_ finds one of the oldest friends that has a minimum degree. This results in the removal of the link between nodes N_p_ and N_b_. The objective in Step 3 is the search for the eldest friend from among the neighbors. If a search of the first hop is unsuccessful, this process will shift to the second hop. That results in making a new link between N_p_ and N_r_. In Step 4, node N_b_ neighbors are selected based on certain priorities. These priorities are created by each node in the network. This step is suitable for real-world scenarios. The priorities are purely based on the frequency of communications and several security parameters, such as trustworthiness, etc. We already surveyed these parameters in our earlier studies [14].

## 5. Experimental Evaluation

### 5.1. Experimental Parameters 

In this paper, we want to study the impact our algorithm has on the objects’ navigability in the network, in order to better understand the concept of object navigability in the SIoT. Initially, we need basic information about the handling of a request for establishing new relations between objects. Network information would be received based on profile, movement, and settings. Since the SIoT is not completely deployed, most experiments are performed in an IoT environment by using several tools; for instance, we used Network X in this study [36]. 

Work presented by Nitte et al. [1] relied on the scale-free Barabasi–Albert (BA) network model. This model is mainly used for the generation of random scale-free networks and is based on preferential attachment (PA) [6]. This network is initialized by using N nodes and m_0_ edges. The BA model has two steps: the first is growth and the second is PA. Initially, the network grows from a few nodes, and later, it adds a number of edges. 

In the PA step, a link is added to an old node that is in proportion to its connectivity. The attachment probability for node w is described in Equation (5):

(5) $$P_{w}=\frac{K_{w}}{\sum_{v\in V}K_{v}}$$

Since the BA model generates a network with low clustering, we decided to use a modified version named the Holme and Kim (HK) model [17]. This HK model is sometimes known as the power law because it obeys the power law. The HK model works in two steps. In the first step, PA is performed, and the second step is triadic formation. Triadic formation is performed by adding an edge between nodes v and w that were added in the previous step for PA. Then, the model adds one more edge from node v to a randomly chosen neighbor of node w. In this model, one incoming node is connected to the maximum number of nodes in the network, and thus it has a greater probability of attaching incoming nodes. The result of the HK model using 15k node edges, m = 5 (averaged over five runs) is described in Table 1. 

In our study, besides one synthetic network, such as the HK model [17], we decided to use and test our algorithm by using some real network data sets, such as Brightkite and Facebook [37]. The parameters we used in our experiments are listed in Table 1. We prefer the HK model due to the performance and power-based settings and, secondly, it has the ability to run all nodes having a huge number of connections, and is similar to those nodes that do not have any connections. 

There are a few drawbacks that we examined in the current data set developed by Nitti et al. in [1]. First, it is not publicly available, and second, it has a low clustering value. Third is the giant component and average path length, with both parameters low at the local level. So, we decided to use Facebook instead of using the SIoT. This is a good starting point for our work and experimentation. 

Thanks to a high clustering coefficient and the scale-free degree distribution property, it makes it possible to find the number of hubs in a network. The first step in this study is to calculate the degree distribution for all data sets, such as Facebook, Brightkite, and the modified HK model. The degree distribution is one of the primary properties of scale-free networks [16] and is calculated by using Equation (6):

(6) $$P\left(k\right)=CK^{-\gamma }$$

**Figure 5:**
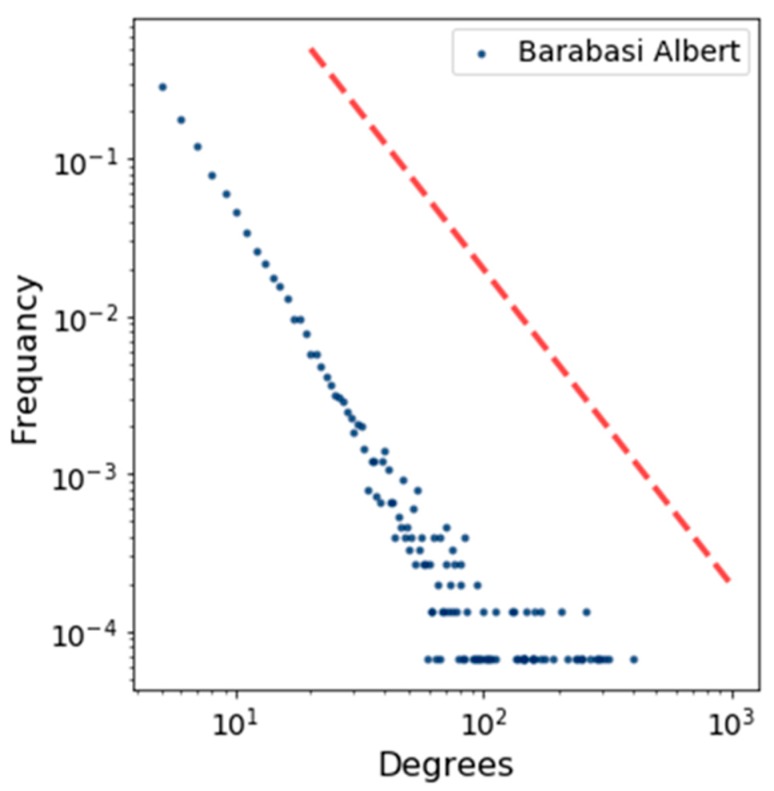
Degree distribution for BA model.

**Figure 6:**
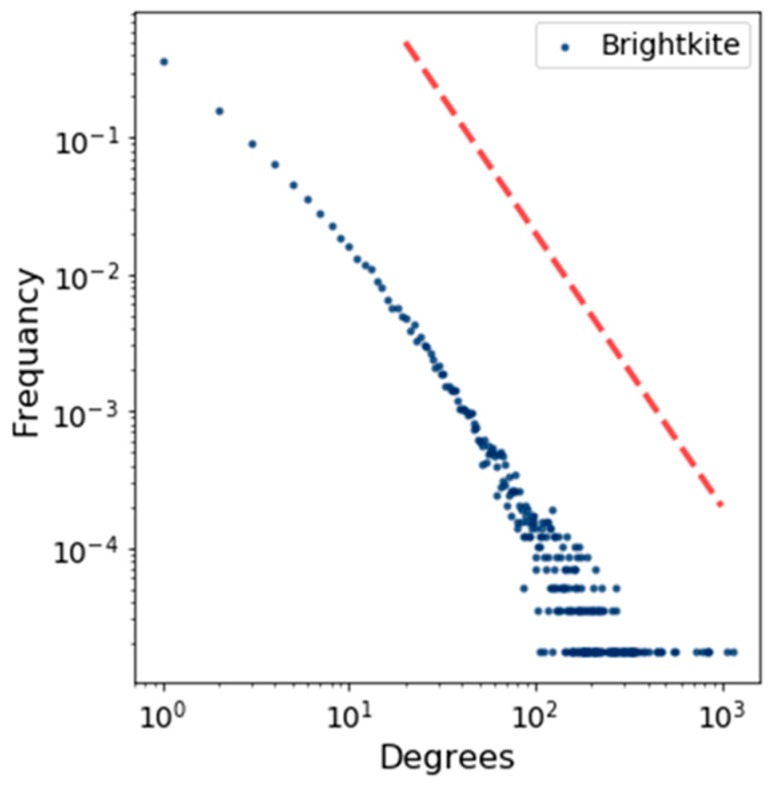
Degree distribution for Brightkite.

**Figure 7:**
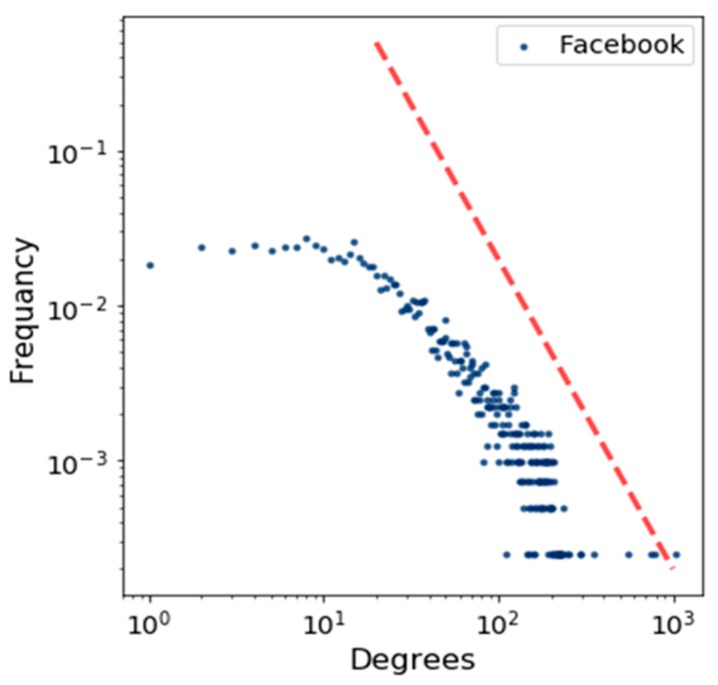
Degree distribution for Facebook.

Figure 5 shows the degree distribution of the BA model by using a log scale in which the power–law function is represented by using a straight red line. This figure indicates how the degrees of a node are distributed. In the same way, we plotted the degree distribution for Brightkite and Facebook in Figure 6 and Figure 7. 

**Figure 8:**
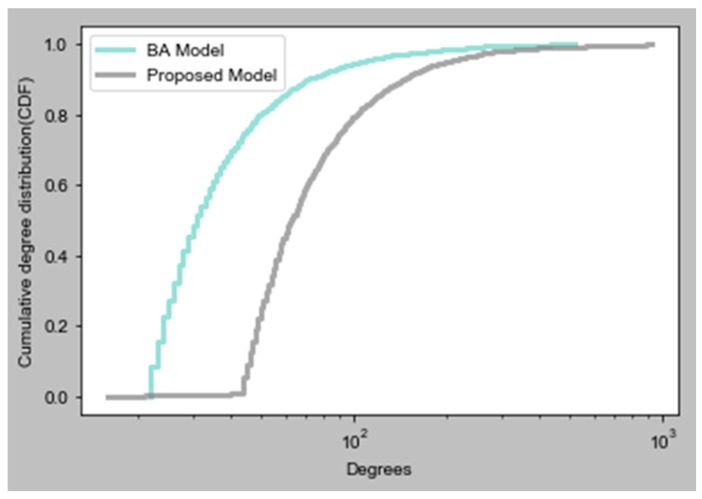
Degree distribution comparison.

Figure 8 demonstrates the degree distribution comparison for the BA model and our modified HK model. In this graph, cumulative degree distribution (CDF) is computed. We plotted both of these functions on a log-x scale by using the Facebook data set. Our objective is to understand how well these models fit into the central part of a distribution. We concluded that the behavior of the BA model is not as good as our model, because our model values are about the median. On a log-log scale, we observed that our model fits the tail of distribution reasonably well, compared to the current model. The graph results demonstrate that our model is best suited to real data sets, such as Facebook and Brightkite. Additionally, in Table 1, we see that the average path length of all three networks is even smaller than the actual network, such as 3.69 [11]. So, plotting of the degree distribution proves it is a good point to start our work and to carefully experiment with these networks by using our proposed algorithm. 

### 5.2. Experimental Results

This section demonstrates our experimental results. We computed these results in terms of local clustering, average path length, clustering coefficient, and giant component. In order to find the connected nodes in the network, first, we needed a standard slicing routine. Slicing is the process of eliminating low-strength edges, sometimes known as weak ties. We performed network analysis based on these slices. Usually, the slicing routine is important for networks, because if we keep all edges (without slicing) we get a distorted view of our network; many algorithms do not discriminate edges by weight. Additionally, if we do not keep the cut-off value in mind, we then get a few problems. If the cut-off is too high, then the network falls apart into small disjointed fragments; if we keep it too low, then the network becomes a hairball, and hence, we cannot analyze the structure of that network. 

**Figure 9:**
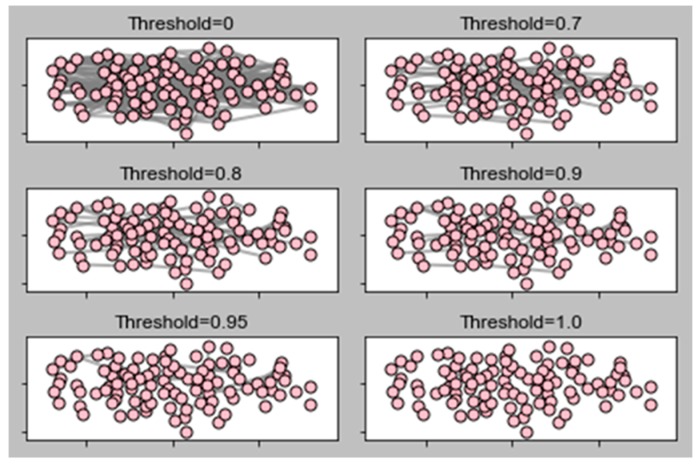
Slicing routine of network.

In order to avoid these situations, we chose a cut-off threshold, t = 1.0, 0.95, 0.9, 0.8, 0.7, and 0, where t controls the density of the entire network. Each edge weight is compared to cut-off threshold t. If edge weight is at or above t, the edge remains in that network; otherwise, it may be erased. Figure 9 shows the slicing routine of our proposed network model of 15k nodes and 75k edges. Due to the huge number of nodes and edges, we cannot differentiate easily; hence, we decided to take a small portion of nodes in a graph. In fact, the objective of this study to reduce the analysis complexity. The experimental results demonstrate a deep understanding of the entire network. We completed a series of tests by applying our algorithm, excluding Step 4. We generated a graph that is primarily based on the parameters in Table 1:

- Suggest first hop.
- Suggest second hop.
- Select and eliminate an old mutual friend.
- Select and eliminate an old mutual friend and also suggest a new friend.
- Eliminate friends based on certain priorities.

The obtained results are described in detail in the next few paragraphs. 

#### 5.2.1. Giant Component

**Figure 10:**
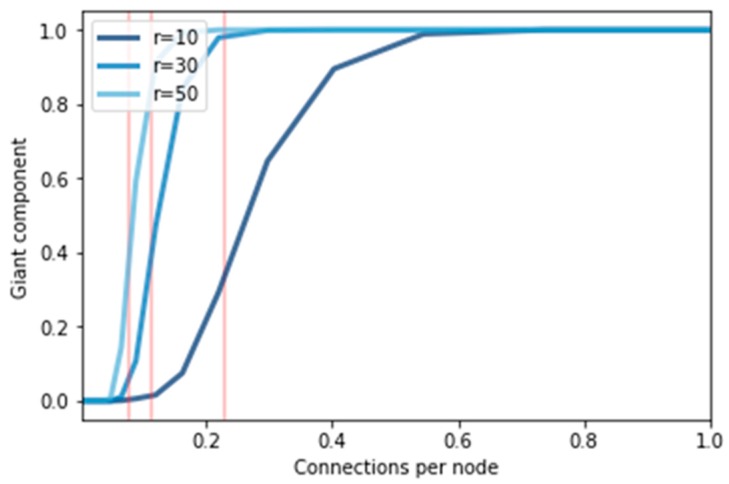
Giant component.

Figure 10 represents the percentage of the giant component after applying different thresholds, i.e., r = 10, 30, and 50. In fact, if we try to reduce the clustering value, we can easily get a large giant component. The justification is that whenever a node having r edges gets a friend request from a node (low connections), then it will simply accept it to the determinant of a node with a higher connection, and it has a high probability of remaining connected to the network. In this figure, we observe that the probability of connected nodes increased by increasing the number of connections per node. The vertical red line in this figure indicates a predicted critical value. We noticed that the giant component gets larger when we use a high value for threshold t. Even in the first hop or second hop, it starts from 0 and will slightly increase up to 100%. For example, when we set threshold r at 10, we observed that it slightly increases from fewer connections. At a certain level, it gets to 100%. Similarly, the highest number for r improves the results, and hence achieves 100%.

#### 5.2.2. Local Clustering Coefficient 

**Figure 11:**
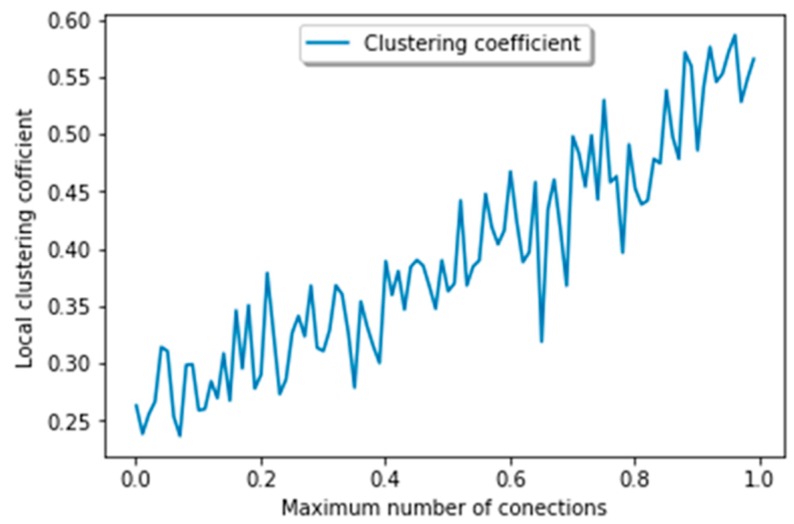
Average clustering coefficient.

Figure 11 shows the result of the average local clustering coefficient. Generally, when the clustering value is 1, it denotes that each node is directly connected to every other node in that network. In this study, our aim is to restrict the number of connections per node, and thus, it results in a lower average clustering coefficient. The number of node connections makes it possible for a network to become a complete graph. There are some other factors, such as suggesting a friend and removal of an old friend instead of one, which are low in degree; the availability of a high number of connections affects the performance of the overall result in the graph. These results can be seen in Figure 11. In this figure, high clustering is due to the dynamic structure, because it can be applied to the first hop or second hop. Usually, average clustering is increased by increasing the number of connections per node. Afterward, it will suddenly decrease to a certain number of connections. The decrement value is in some ways negligible and admissible, since it is helpful in getting a higher giant component (as discussed in the above section). The obtaining of a higher clustering coefficient clearly indicates the effectiveness of our algorithm, and additionally proves that it is moving towards a clique.

#### 5.2.3. Average Path Length 

**Figure 12:**
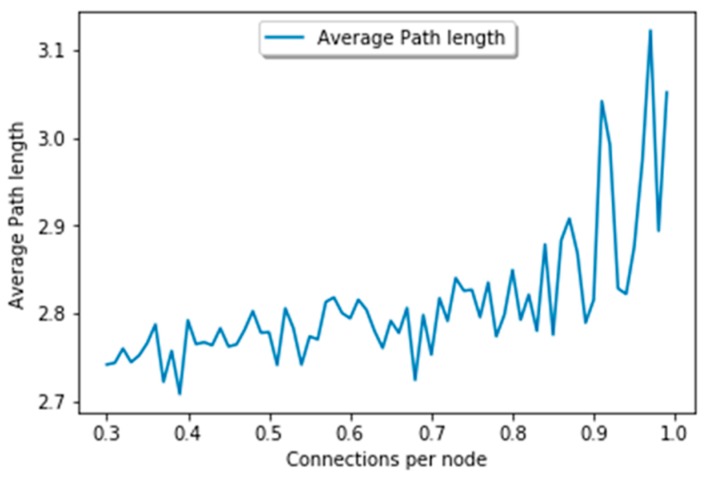
Average path length.

The average path length of our graph is shown in Figure 12. In this figure, we can clearly understand that it has a shorter path length and a high degree. This means that the number of hops between two nodes reduces slightly. The maximum path length of our graph is 3. We carefully examined the current work and found that the maximum path length is from 4 to 4.5. The existence of a shorter path in this graph proves the performance of our algorithm. The low average path length is in fact due to the dynamic threshold adjustment and the ability of our algorithm to manage and create long-distance relationships. Moreover, the search latency is decreased with a decrease in path length, and hence our algorithm behaves efficiently compared to the earlier heuristics. 

After completing all experiments by using our algorithm, we can say that our proposed algorithm is helpful in attaining high network navigability, and it later results in an increase in the giant component. Our algorithm is suitable for first or second hops. Finally, applying our proposed algorithm to data sets did not pull any parameters down.

#### 5.2.4. Execution Time

**Figure 13:**
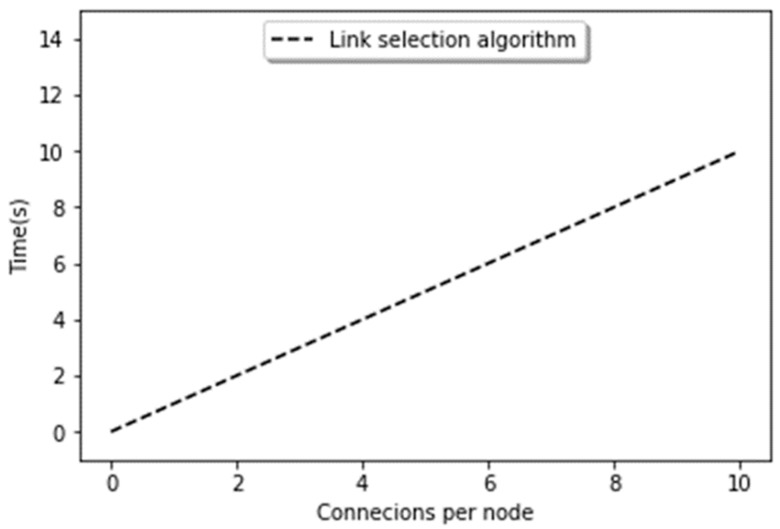
Execution time.

Figure 13 shows the overall execution time of our proposed algorithm. Usually, the execution time totally depends upon the number of hubs, the number of receiving nodes, and the method that is used for the representation of data in the network. In our case, linear dependence is determined by the value of ‘r’. If the modified power law is strongly unbalanced, on average, it will take a few steps for each search. The execution, in fact, depends upon the preferential attachment algorithm, which requires O(N) iterations on average, and on the number of iterations selected, which is directly proportional to the number of connections in r. The result reflects the fact that as the connections are growing, the execution time increases. The number of connections grows over time, and it results in the increase of execution time. We tested the algorithm only up to r = 10, 30, and 50. It should be noted that the proposed algorithm is based on preferential attachment and triadic closure which are essential parts of complex networks, thus can be applied in realistic IoT applications.

## 6. Conclusion and Future Work

This paper presented the issue of link selection in the SIoT, where objects establish friendship links with each other, thus creating a social network of objects. First, we analyzed network navigability in social networks through example-based scenarios and simulation as this is important for service discovery. Second, we proposed an advanced algorithm that has a threshold value and can be adjusted dynamically on the basis of the number of hubs in a network. Our algorithm performs service discovery, adding new links and removing old mutual friends in a decentralized manner. Once discovery through one friend is finished, the search through a new friend in the second hop is performed. This phenomenon helps one friend to connect with a high-degree friend, thus, results in an increase in object navigability in the network. Our algorithm has a different impact on network structures by utilizing small-world network properties, such as giant component, local clustering coefficient, and path length. The experimental results prove that our algorithm performs well in terms of attaining larger clustering, shorten path length and finally obtains a higher giant component. It should be noted that the proposed algorithm is based on preferential attachment and triadic closure which are essential parts of complex networks, thus, can be applied in realistic IoT applications. As future work, we plan to focus on combining similarity features in the same scenario using trustworthiness.

## Figures and Tables

**Table 1:** Parameters of Barabasi–Albert, Brightkite, and Facebook.

Parameters	HK Model	Brightkite	Facebook
Nodes	15,000	58,228	40,399
Edges	75,000	214,078	88,234
Average degree	9.99	7.35	43.69
Average clustering coefficient	0.03	0.1723	0.602
Average path length	3.781	0.247	3.686
Network diameter	5	14	8
Giant component	100%	92%	85%
